# Ameliorative effect of folic acid and vitamin B12 against Ivermectin-induced hepatotoxicity, renal toxicity, oxidative stress and immunohistochemical changes in male albino rats

**DOI:** 10.1038/s41598-025-23047-2

**Published:** 2025-10-31

**Authors:** Rana A. Ali, Yahia A. Amin, Zeinab A. Mar’ie, Asmaa M. Mosa, Seham A. Mobarak

**Affiliations:** 1 Department of Zoology, Faculty of Science, South Valley University, Qena, Egypt; 2 Department of Theriogenology, Faculty of Veterinary Medicine, Aswan University, Aswan, Egypt; 3 Department of Science, Faculty of Education, Hurghada University, Hurghada, Egypt

**Keywords:** Ivermectin, Folic acid, Vitamin B12, Hepatotoxicity, Renal toxicity, Oxidative stress, Immunohistochemical changes, Male albino rats, Biochemistry, Cell biology, Chemical biology, Drug discovery, Physiology, Zoology

## Abstract

Since its remarkable discovery 35 years ago, Ivermectin (IVM) has remained one of the most crucial medications for treating parasitic infections in both human and animal medicine. Ivermectin was recommended in high doses for treatment of SARS-CoV-2, severe acute respiratory syndrome coronavirus 2 and as a part of feed in fish farms that exposed the treated animal or human for risk of its toxicity. It can cause hepatotoxicity, renal toxicity and other side effects, which may require prompt intervention for removal. Therefore the current study aimed to investigate the potential protective effects of folic acid (FA) and vitamin B12 (Vit.B12) against IVM-induced hepatotoxicity and renal toxicity in rats after prolonged high-dose exposure. 28 male Albino rats were divided into four groups of seven rats for each group. The first group is the control group (Ctrl group) received distilled water, the second group was subcutaneously injected with IVM (IVM group), the third group was subcutaneously injected with IVM and orally administered FA (IVM + FA group), and the fourth group was subcutaneously injected with IVM and intramuscular injection of Vit.B12 (IVM + Vit.B12 group). After 30 days of the experiment, blood samples were collected for biochemical analysis while liver and kidney tissue specimens were collected for oxidative analysis and histological investigation. In addition, immunohistochemistry assays of caspase3, BAX protein, Bcl2 protein and PCNA were checked. Results revealed that IVM group displayed significant increase in the liver enzymes (AST and ALT) associated with decreased albumin, total protein and alkaline phosphatase in the serum. All kidney parameters were found significantly increased. In addition, significant increase was observed in malondialdehyde combined with significant decrease of catalase, total antioxidant capacity and superoxide dismutase in the IVM group. IVM group showed histological and immunohistochemical changes in the liver and the kidney compared to the control group. Immunoexpression of caspase3 and BAX were enhanced after administration of IVM while Bcl2 protein and PCNA were negatively expressed. All of these above mentioned parameters were almost returned to normal levels after administration of FA and Vit.B12 associated with improvement in liver and kidney function. These findings highlight the ameliorative effects of FA and Vit.B12, emphasizing the necessity of their co-administration with IVM to maximize its therapeutic benefits-including promoting food security-while minimizing its harmful effects on the liver and kidneys during treatment protocols.

## Introduction

Ivermectin (IVM) is a widely used antiparasitic drug in animals and human^1^. It is used as antiviral^2^, antibacterial^3^ and anticancer^4^. As IVM is a safe drug^5^, it was used in high concentrations (6 mg/kg) as antiviral drug against SARS-CoV-2 in vitro^6,7^ and as a part of feed in fish farms^8^. Therefore, variant studies evaluated its toxicity at high dose^9^, and showed that IVM suppress coronavirus 2 in-vitro with high un-recommended doses^10^. However the high dose of nebulized IVM can damage lung architecture^11^.

Additionally, the low dose (0.19 g/kg) of IVM microemulsion alters the liver function when it was used for a long duration^12^, causing changes in the liver enzymes, kidney biomarkers^13^ and induce oxidative stress^14^. Furthermore, the negative impact of IVM not only limited to the animals or humans but extends to plants^15^. Thus, looking for a method or therapy that alleviates these drawbacks of IVM is essential.

Oxidative stress can induce a variety of harmful effects in different body systems and play a role in the mechanisms and pathogenesis of various diseases. Therefore, several researchers have focused on studying oxidative stress^16,17^ and the antioxidant mechanisms that counteract it ^18–21^. Ivermectin has been shown to induce oxidative stress in the tissues of North African catfish^22^. There is clear evidence linking drug-induced oxidative stress as a mechanism of toxicity in various tissues^23^.

Folic acid (FA), a multifunctional vitamin B9, is recognized for its antioxidant and anticancer properties, as well as its roles in cardiovascular and neuroprotective health^24^. Notably, its antioxidant activity surpasses that of vitamins C and E^25^. Folic acid has a good impact on liver through reduction of liver enzymes (ALT and AST), improve liver steatosis in non-alcoholic liver disease^26^ and exerted anti-inflammatory effect on the liver of alcoholic liver disease^27^. In kidney, FA treated renal fibrosis^28^. Vit.B12, as a vitamin, shares FA the antioxidant property^13^. It provide protection against liver necrosis caused by methotrexate^29^, and reduces levels of creatinine and uric acid after nephrotoxicity by the same component^30^.

The present study hypothesizes that administration of folic acid (FA) and vitamin B12 (Vit.B12) can provide protective effects and mitigate the toxic impact of high doses of ivermectin (IVM) on hepatic and renal organs. The aim of this study was to investigate the ameliorative effects of FA and Vit.B12 against the harmful effects of high IVM doses on liver and kidney tissues. Additionally, the study evaluated both the combined and independent effects of high-dose IVM on body weight development in the tested rats. Histopathological, immunohistochemical changes, oxidative stress levels, and antioxidant enzyme activities were also assessed in liver and kidney tissues.

## Materials and methods

### Drugs

Ivermectin (IVM) (Bomectin injection), was produced by Bayer (Germany company). Folic acid (FA) tablets, produced by El-Nile Company for pharmaceuticals and chemical industries, dissolved in adequate amount of distilled water. Vit.B12 (Depovit B12), produced by Amriya for pharmaceutical industries-Egypt, Vit.B12 ampoule dissolved in distilled water to facilitate injection of the obtained dose.

### Animals and treatments

All procedures were carried out according to the guide that was approved by the Ethics Committee of the Faculty of Science, South Valley University, Egypt (approval number 005/06/24). All methods were performed in accordance with the relevant guidelines and regulations. The research was done in accordance with the ARRIVE criteria.

Twenty-eight adults’ male Wistar albino rats aged between 13 and 15 weeks and weighing an average of 200 ± 20 g were obtained from the Laboratory Animal Housing Unit of the Faculty of Veterinary Medicine at South Valley University in Egypt. The rats were housed in plastic cages and were maintained on commercial standard diet with water ad libitum. Rats were acclimatized for two weeks before the experiment.

Rats were divided into four equal groups at random (7 rats /group) and received their treatments daily for 30 consecutive days. Group 1 was the control group (ctrl group) where the rats were orally administered saline solution (Nacl 0.9%) by gavage. Group 2 was the ivermectin group (IVM group) where the rats were administered IVM (5 mg/kg/day)^31^, by subcutaneous injection. Group 3 was IVM + FA group where the rats were co-administered IVM by subcutaneous injection with the same previously mentioned dose and orally administrated FA at a dose of 20 mg/kg^32^. Group 4 was IVM + Vit.B12 group where the rats were co-administered IVM by subcutaneous injection with the same previously mentioned dose and intramuscular injection of Vit.B12 (50 mcg)^33^.

### Euthanasia procedure

At the end of the experimental period, all animals were humanely euthanized for the purpose of sample collection. Rats were individually placed in an induction chamber and anesthetized by inhalation of 3% isoflurane delivered in 100% oxygen at a flow rate of 1–2 L/min. The animals were monitored closely for signs of deep anesthesia, including the absence of pedal withdrawal and corneal reflexes, to ensure full unconsciousness. Once anesthesia was confirmed, euthanasia was carried out through decapitation. In cases where rapid tissue harvesting was critical, decapitation was performed immediately following deep anesthesia to ensure the animals did not regain consciousness. All procedures were performed by trained personnel and were in compliance with institutional and national ethical guidelines for animal experimentation. After euthanasia, relevant tissues and organs were rapidly harvested^34^.

### Blood sampling and biochemical analysis

Blood samples were obtained via the retro-orbital sinus and collected into plain tubes for biochemical analysis. The blood was allowed to coagulate, and then centrifuged at 3000 rpm for 15 min to separate the serum, which was used for biochemical assessments. Aspartate transaminase (AST) and alanine transaminase (ALT) were analyzed using kinetic measurements, while alkaline phosphatase (ALP), total protein (TP), albumin (ALB) and cholesterol were analyzed colorimetrically. On the other hand, kidney parameters such as creatinine and uric acid (UA) were analyzed colorimetrically, while urea and BUN were measured kinetically. The kits supplied by Roche, Egypt, were used for the analysis of these parameters.

### Oxidative stress measurements

Specimens of liver and kidney were obtained from the dissected rat and stored at – 80 °C for the analysis of oxidant and antioxidant biomarkers. Malondialdehyde (MDA) was measured using the Rat Malondialdehyde ELISA Kit. Superoxide dismutase (SOD) was measured using the Rat Superoxide Dismutase ELISA Kit (Catalog No.CSB-E08555r). Total antioxidant capacity (TAC) was assessed using the Rat Total Antioxidant Status ELISA Kit (Cat. No MBS1600693). Catalase (CAT) levels were determined using the Rat Catalase (CAT) ELISA Kit (Cat No. MBS2600683). All kits employed a quantitative competitive ELISA technique.

### Histopathological examination

Specimens of liver and kidney were obtained from the dissected rat and rapidly fixed in 10% formalin for 24 h. The tissues were then transferred to 70% ethyl alcohol, followed by ascending grades of alcohol for dehydration, and cleared with xylene. Impregnation was done in pure soft paraffin at 50 °C for 2 h, followed by embedding in hard paraffin. Sections of 3–5 microns in thickness were cut from each block using a microtome and stained with hematoxylin and eosin to study the general histological structure^35^.

### Immunohistochemical investigation

The selected sections were deparaffinized in xylene for 20 min, rehydrated in downgraded alcohol (100%, 80%, 70%, and 50%) 2 min for each, then rinsed in distilled water. Tissue sections were incubated in 0.5% hydrogen peroxide/methanol for 10 min to block endogenous peroxidase activity followed by washing twice in phosphate buffer saline (PBS)^36^. Antibodies of caspase, proliferating cell nuclear antigen (PCNA), B-cell lymphoma 2 (Bcl2), Bcl-2-associated X-protein (BAX) were incubated in these manipulated tissues for detection of immunohistochemical changes.

For quantitative evaluation of immunohistochemical staining, digital images of liver and kidney sections were analyzed using ImageJ software. The *Integrated Density (IntDen)* value was measured for each marker (Caspase-3, BAX, Bcl-2, and PCNA). IntDen represents the sum of pixel values within the selected stained area and reflects both the extent and intensity of immunopositivity. For each tissue, at least five randomly selected fields per section were analyzed, and the mean IntDen values were calculated. These quantitative data were used for statistical comparison among groups.

### Statistical analysis

The variability degree of results was expressed as Mean ± Standard deviation (Mean ± S.D). The data were statistically analyzed by One-Way ANOVA analysis of variance (Prism Computer Program) and the least significant difference (L.S.D) was used to test the difference between treatments. Results were considered statistically significant when *P* < 0.05.

## Results

Changes in the liver profile of the control group and the other groups treated with ivermectin (IVM) alone or in combination with folic acid (FA) and/or vitamin B12 (Vit.B12) are presented in Table 1. Ivermectin administration significantly increased AST and ALT activities compared to the control group (*P* < 0.05), while ALP, ALB, and TP levels were significantly decreased. Co-administration of FA with IVM (IVM + FA group) significantly reduced AST, ALT, and ALP levels compared to the IVM group, while TP levels significantly increased. Administration of Vit.B12 in combination with IVM resulted in a non-significant change in ALT, ALP, and ALB levels compared to the IVM group; however, AST was significantly decreased and TP was significantly increased.

**Table 1:** Changes in liver profile of the control group and experimental groups treated with Ivermectin alone or in combination with folic acid and/or vitamin B12 in adult male albino rats.

Parameters	ALT (U/L)	AST (U/L)	ALP (U/L)	ALB (U/L)	TP (U/L)
Ctrl group	39.7 ± 2.5^b^	103.9 ± 5.9^b^	218.0 ± 3.6^a^	3.7 ± 0.20^a^	5.9 ± 0.2^a^
IVM group	47.5 ± 1.7^a^	151.8 ± 3.5^a^	150.3 ± 6.5^b^	2.6 ± 0.14^b^	5.53 ± 0.1^b^
IVM + FA group	32.4 ± 7.9^b^	111.8 ± 3.1^b^	111.3 ± 6.4^c^	2.8 ± 0.09^b^	5.98 ± 0.3^a^
IVM + Vit. B12 group	44.8 ± 3.0^a^	111.6 ± 17.0^b^	160.8 ± 6.0^b^	2.6 ± 0.07 ^b^	5.8 ± 0.1^a^

Values are expressed as means ± S.D. Values with different superscript (a, b and c) in the same column are significantly different at *P* < 0.05. Ctrl group: Control group; IVM group: Ivermectin group; IVM + FA group: Ivermectin + Folic acid group; IVM + Vit. B12 group: Ivermectin + Vit. B12 group; ALB: Albumin; TP: Total protein.

Changes of kidney profile were shown in Table 2, IVM significantly increased all assessed kidney parameters compared to CTRL group. Folic acid administration significantly reduced the elevation of those kidney parameters compared to IVM group. Vit.B12 administration significantly reduced the elevation of the kidney parameters compared to IVM group except for BUN and uric acid (UA) that exhibit non-significant change.

**Table 2:** Changes in kidney profile of the control group and experimental groups treated with Ivermectin alone or in combination with folic acid and/or vitamin B12 in adult male albino rats.

Parameters	Creatinine (mg/dl)	Urea (mg/dl)	BUN (mg/dl)	UA (mg/dl)
Ctrl group	0.46 ± 0.11^b^	35.17 ± 02^c^	19.57 ± 4.79^b^	2.1 ± 0.12^b^
IVM group	1.2 ± 0.1^a^	134.5 ± 6.5^a^	49.25 ± 13.9^a^	3.4 ± 0.45^a^
IVM + FA group	0.71 ± 0.1^b^	50.33 ± 2.7^c^	24.20 ± 2.9^b^	2.5 ± 0.5^b^
IVM + Vit. B12 group	0.54 ± 0.04^b^	90.00 ± 4.3^b^	47.29 ± 9.6^a^	3.1± 0.19^a^

Values are means ± S.D. Values with different superscript (a, b and c) in the same column are significantly different at *P* < 0.05. Ctrl group: Control group; IVM group: Ivermectin group; IVM + FA group: Ivermectin + Folic acid group; IVM + Vit. B12 group: Ivermectin + Vit. B12 group; BUN: Blood urea nitrogen; UA: Uric acid.

Changes of oxidative biomarkers and antioxidant enzymes in liver and kidney tissues were shown in Tables 3 and 4, respectively. Concentrations of malondialdehyde (MDA) in the liver and kidney of IVM-group significantly increased rather than those of the control group (*P* < 0.05). The other two treated groups with FA and Vit.B12 displayed significant decrease of the level of MDA in the liver and kidney tissues. The antioxidants; CAT, SOD and TAC were significantly decreased in response to IVM compared to their respective controls in both organs. Administration of FA and Vit.B12 with IVM significantly increased the activities of previously mentioned antioxidants (*P* < 0.05).

**Table 3:** Changes of oxidative biomarkers and antioxidant enzymes in liver tissues of male albino rats of the control group and experimental groups treated with Ivermectin alone or in combination with folic acid and/or vitamin B12 in adult male albino rats.

Parameters	CAT (ng/ml)	SOD (U/ml)	MDA (nmol/ml)	TAC (U/ml)
Ctrl group	130.6 ± 1.6^a^	57.52 ± 1.1^a^	7.21 ± 0.3^b^	39.65 ± 1.6^a^
IVM group	79.97 ± 3.4^b^	35.50 ± 2.1^b^	18.02 ± 1.5^a^	29.61 ± 1.3^b^
IVM + FA group	106.2 ± 3.83^a^	89.62 ± 2.6^a^	9.95 ± 0.7^b^	44.55 ± 2.2^a^
IVM + Vit. B12 group	96.18 ± 2.2^a^	81.00 ± 2.6^a^	7.82 ± 1.1^b^	61.52 ± 4.1^a^

Values are means ± S.D. Values with different superscript (a, b and c) in the same column are significantly different at *P* < 0.05. Ctrl group: Control group; IVM group: Ivermectin group; IVM + FA group: Ivermectin + Folic acid group; IVM + Vit. B12 group: Ivermectin + Vit. B12 group;

**Table 4:** Changes of oxidative biomarkers and antioxidant enzymes in kidney tissue of male albino rats of the control group and experimental groups treated with Ivermectin alone or in combination with folic acid and/or vitamin B12 in adult male albino rats.

Parameters	CAT (ng/ml)	SOD (U/ml)	MDA (nmol/ml)	TAC (U/ml)
Ctrl group	134.4 ± 2.2^a^	51.09 ± 3.02^b^	6.45 ± 0.5^c^	36.00 ± 1.7^b^
IVM group	70.45 ± 4.6^b^	33.62 ± 2.4^c^	18.82 ± 0.5^a^	26.95 ± 1.9^c^
IVM + FA group	104.3 ± 5.3^a^	93.47 ± 4.9^a^	11.24 ± 0.8^b^	43.17 ± 1.5^a^
IVM + Vit. B12 group	99.24 ± 1.0^a^	72.88 ± 3.6^a^	9.44 ± 0.7^b^	55.35 ± 3.08^a^

Values are means ± S.D. Values with different superscript (a, b, c or d) in each row are significantly different at *P* < 0.05. Ctrl group: Control group; IVM group: Ivermectin group; IVM + FA group: Ivermectin + Folic acid group; IVM + Vit. B12 group: Ivermectin + Vit. B12 group; CAT: Catalaze; SOD: Superoxide dismutase;

### Histopathological and immunohistochemical findings of the liver

**Fig. 1:**
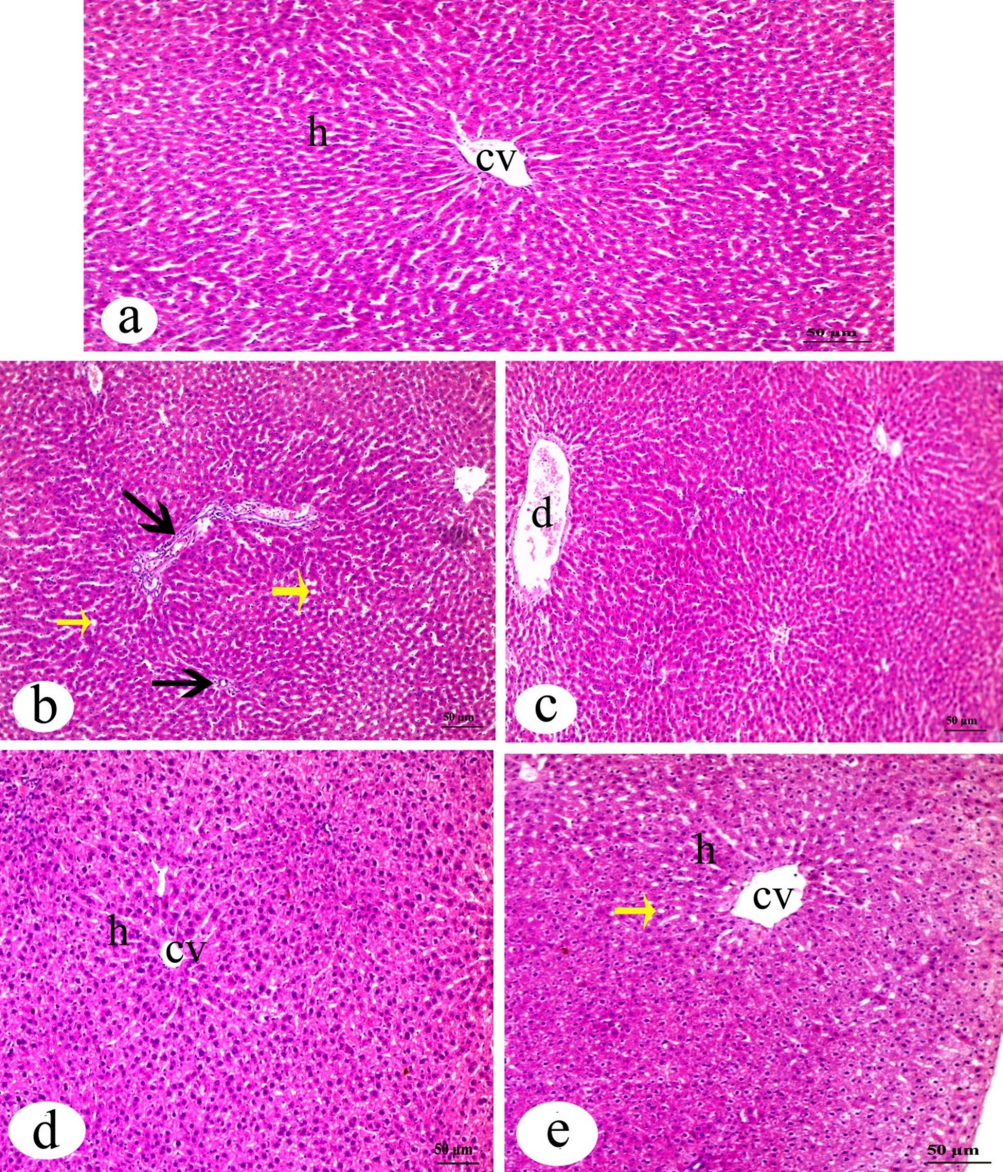
Photomicrograph of H&E-stained liver sections from the indicated groups: (**a**) control male rat, showing the normal appearance of liver indicating central vein (cv) and hepatocytes (h); (**b**) and (**c**) IVM group, revealing loss of hepatic architecture, multiple ballooned hepatocytes (yellow arrow), inflammatory inflammation (black arrow), and dilatation of blood vessels (**d**); (**d**) IVM + FA group: showing hepatic tissues with less cell infiltration and appearance of healthy architecture of liver; (**e**) IVM + Vit.B12 group: demonstrating healthy architecture appearance similar to control (H&E Bar = 50 μm).

The histology of liver was found normal in the control group with a typical structure of central vein and hepatocytes as shown in Fig. 1. IVM group showed a loss of hepatic architecture, multiple ballooned hepatocytes with presence of inflammation, and dilatation of blood vessels. Treatment with FA and Vit.B12 restored normal tissue histology nearly similar to the control group (Fig. 1).

**Fig. 2:**
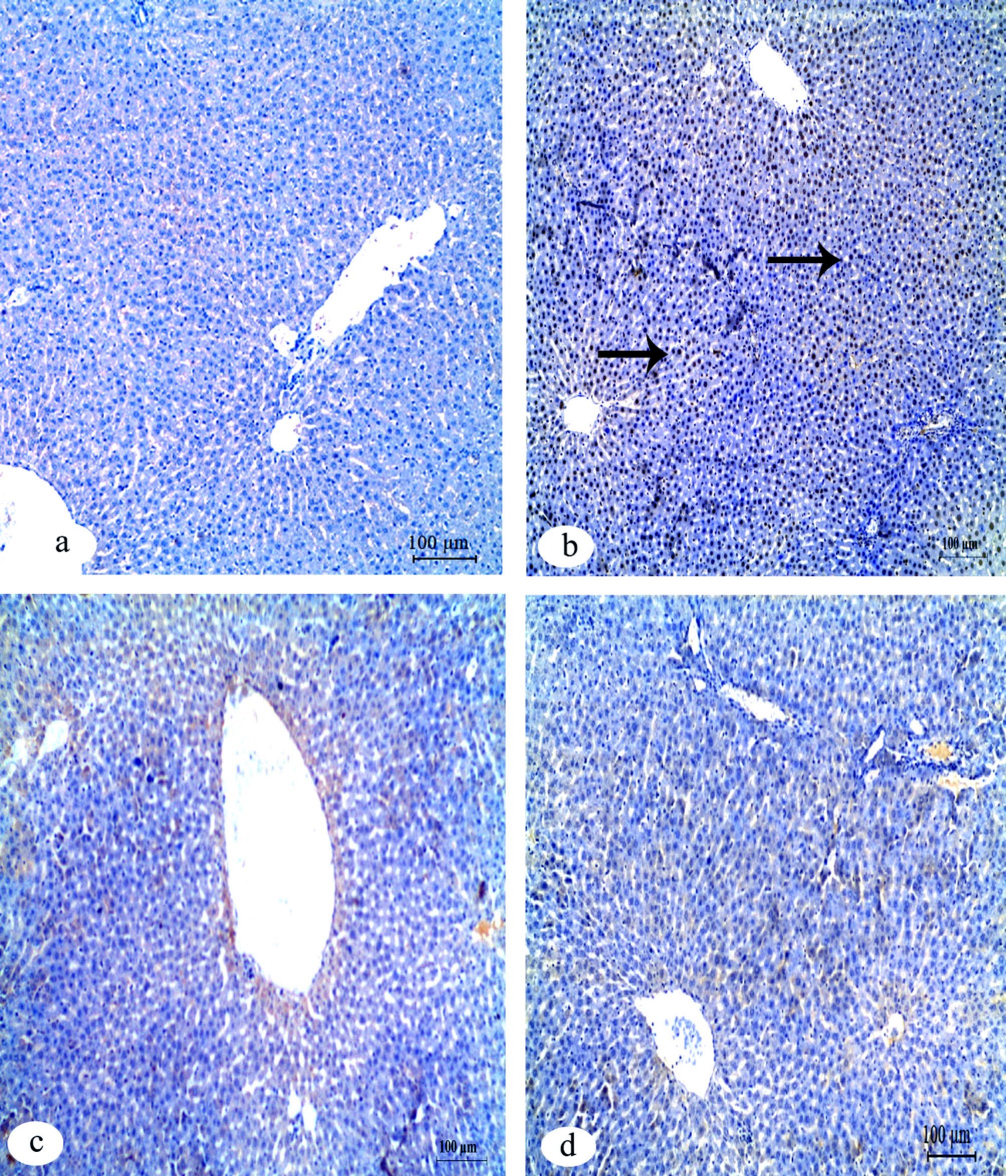
Immunohistochemical staining for cleaved caspase-3 expression in liver tissue sections of male albino rats. (**a**) Control group showing negative immunoreactivity. (**b**) IVM-treated group showing strong positive staining indicating increased apoptotic activity. (**c**) IVM + folic acid-treated group exhibiting moderate reduction in caspase-3 immunoreactivity compared to the IVM group. (**d**) IVM + vitamin B12-treated group showing minimal to absent immunoreactivity, approaching levels observed in the control group. Black arrows indicate positive reactions. Scale bar = 100 μm.

**Fig. 3:**
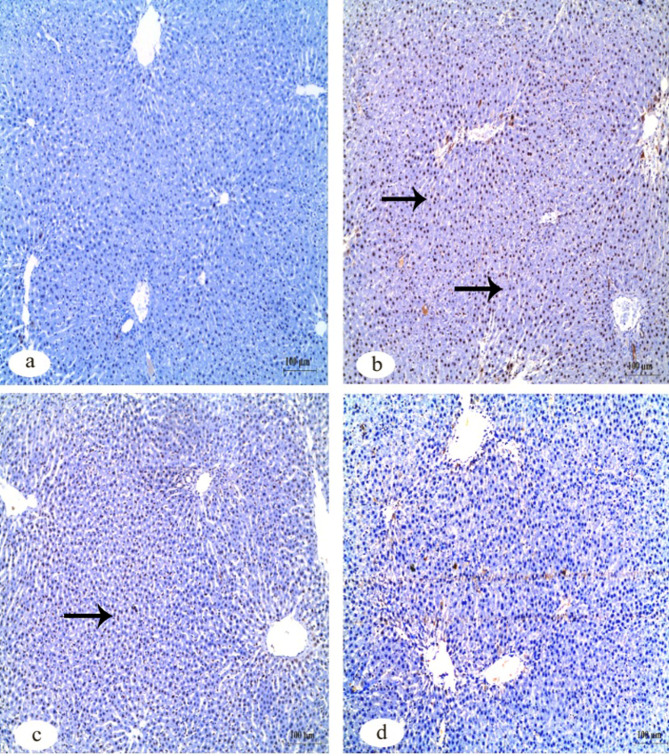
Immunohistochemical staining for BAX expression in liver tissue sections of male albino rats. (**a**) Control group showing negative immunoreactivity. (**b**) IVM-treated group showing strong positive staining indicating increased apoptotic activity. (**c**) IVM + folic acid-treated group exhibiting moderate reduction in Bax immunoreactivity compared to the IVM group. (**d**) IVM + vitamin B12-treated group showing minimal to absent immunoreactivity, approaching levels observed in the control group. Black arrows indicate positive reactions. Scale bar = 100 μm.

**Fig. 4:**
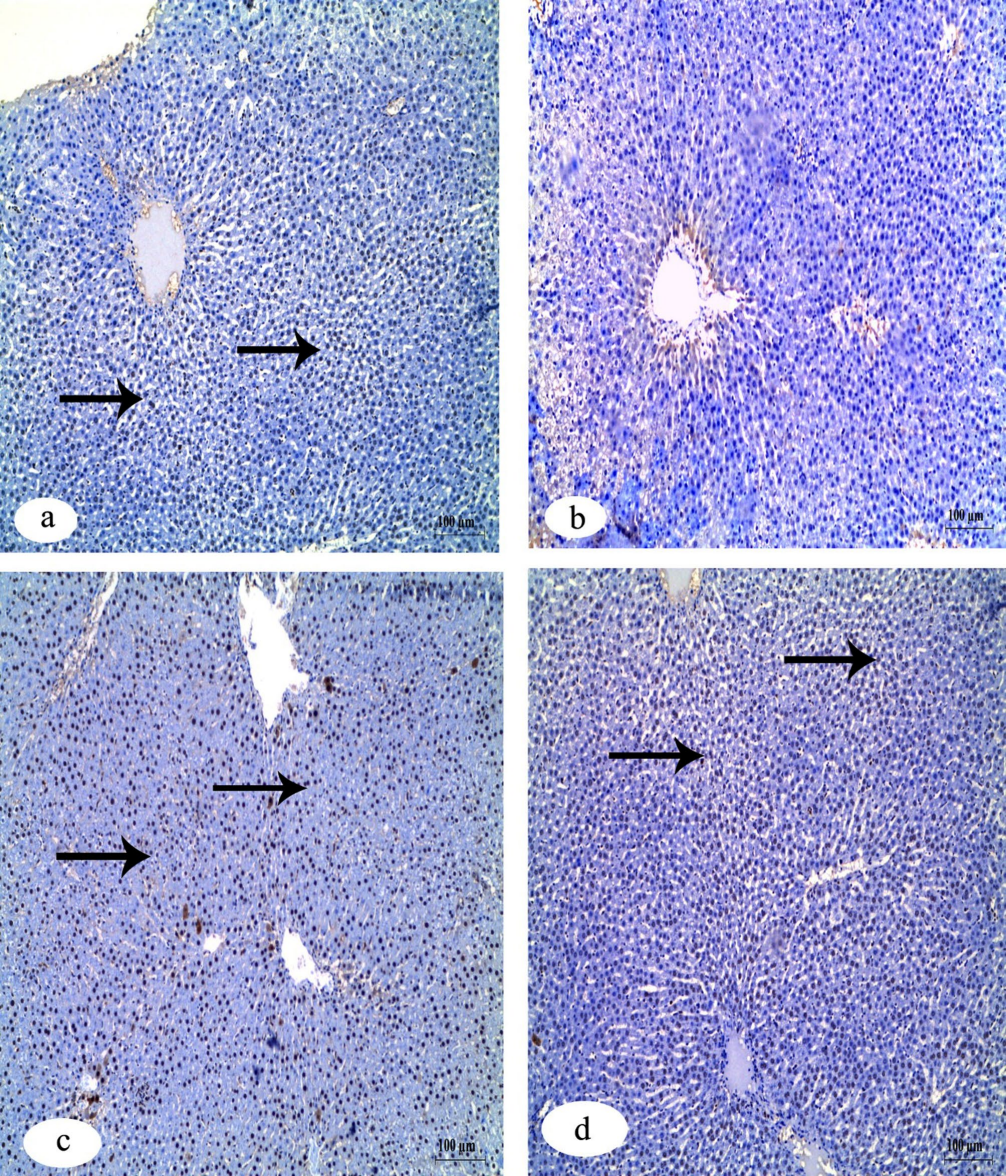
Immunohistochemical staining for Bcl-2 protein expression in liver tissue sections of male albino rats. (**a**) Control group showing strong positive Bcl-2 immunoreactivity. (**b**) IVM-treated group demonstrating a marked loss of Bcl-2 expression, indicating reduced anti-apoptotic activity. (**c**) IVM + folic acid-treated group showing partial restoration of Bcl-2 expression. (**d**) IVM + vitamin B12-treated group exhibiting positive Bcl-2 staining comparable to the control. Black arrows indicate positive reactions. Scale bar = 100 μm.

**Fig. 5:**
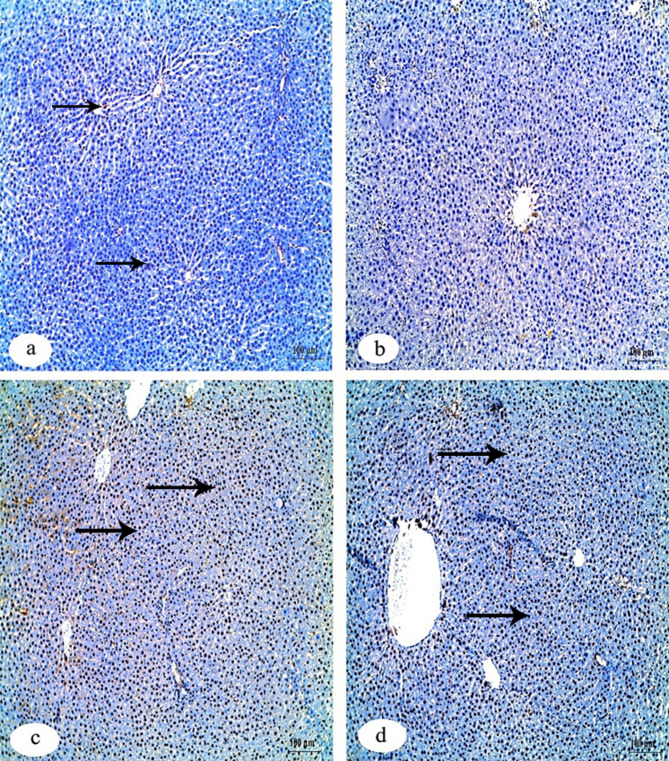
Immunohistochemical staining for PCNA expression in liver tissue sections of male albino rats. (**a**) Control group exhibiting strong positive PCNA immunoreactivity, indicating active cell proliferation. (**b**) IVM-treated group showing markedly reduced PCNA expression, suggesting impaired hepatocellular regeneration. (**c**) IVM + folic acid-treated group showing moderate restoration of PCNA-positive nuclei. (**d**) IVM + vitamin B12-treated group showing PCNA expression levels comparable to the control group. Black arrows indicate positive reactions. Scale bar = 100 μm.

Immunohistochemical investigation of caspase-3 in liver tissue of the control group, IVM + FA group and IVM + Vit.B12 group revealed negative reaction compared to its positive reaction in IVM group (Fig. 2). For immunohistochemical investigation of BAX protein in the liver tissue, results showed expression of a negative reaction in the control group, while the group of IVM displayed a positive reaction. The two groups of FA and Vit.B12 have a weak reaction in BAX protein (Fig. 3). Immunohistochemical investigation of Bcl-2 and PCNA in the liver tissues revealed positive reaction in the control group compared with negative reaction in the IVM group. While, the groups of IVM + FA and IVM + Vit.B12 revealed a positive reaction (Figs. 4 and 5, respectively).

**Fig. 6:**
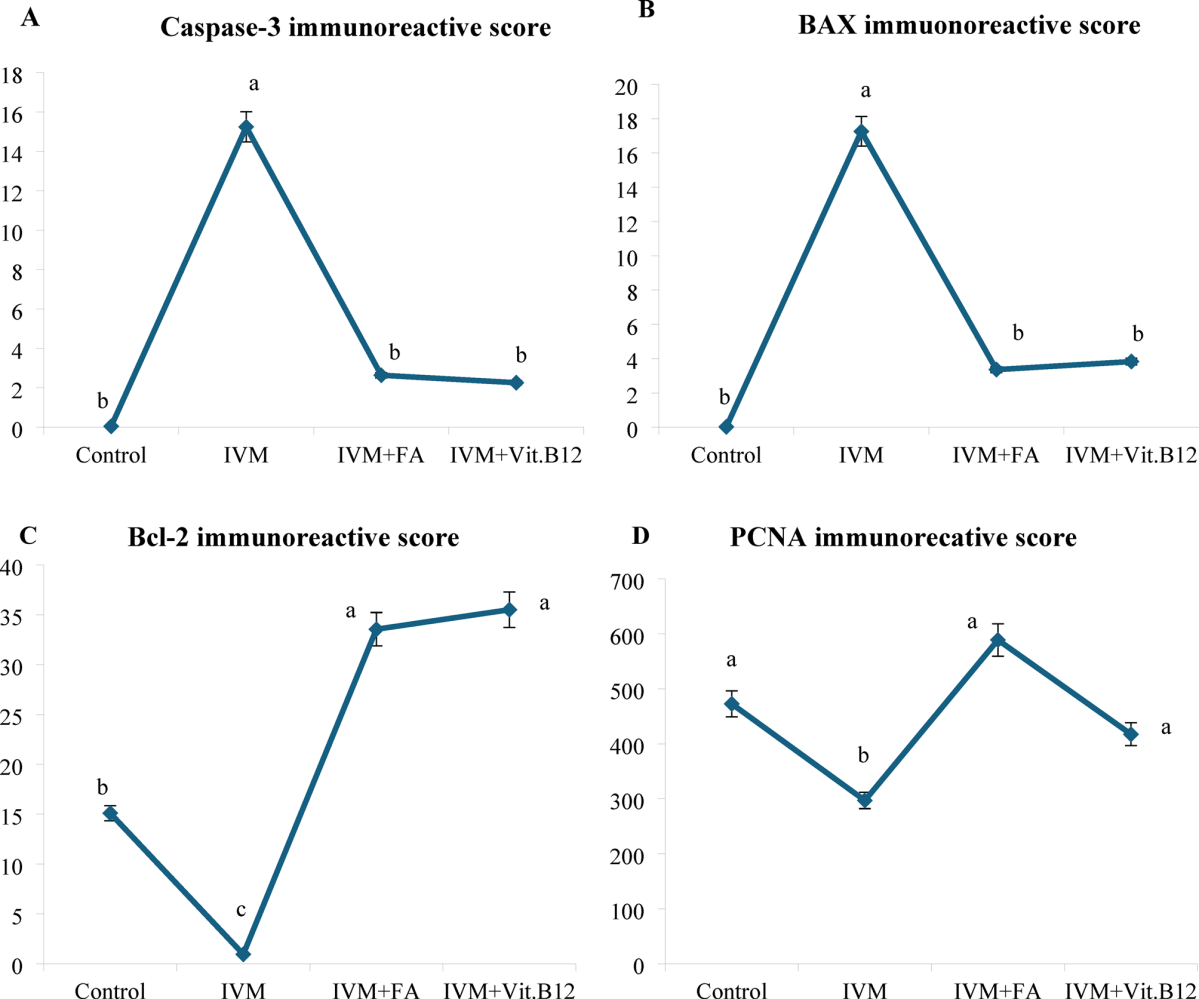
Immunoreactive score of (**A**) Caspase-3, (**B**) Bax, (**C**) Bcl-2 and (**D**) PCNA in liver sections of male rats from different treatment groups. Values are expressed as means ± S.D. Values with different superscript (a, b and c) are significantly different at *P* < 0.05. Control: Control group; IVM: Ivermectin group; IVM + FA: Ivermectin + Folic acid group; IVM + Vit. B12: Ivermectin + Vit. B12 group.

Table 5 shows quantitative analysis of Integrated Density (IntDen) Values for Caspase-3, BAX, Bcl-2 and PCNA expressions in the liver tissues. Results of the IntDen analysis of cleaved caspase-3 and BAX expressions in the liver sections revealed a marked increase in the apoptotic activity in the IVM-treated group compared to the control. The IVM group showed strong cytoplasmic immunoreactivity, as indicated by the intense brown staining and significantly elevated IntDen score (Fig. 6A and B). In contrast, co-treatment with folic acid (IVM + FA) or vitamin B12 (IVM + Vit.B12) resulted in noticeably reduced cleaved caspase-3 and Bax expressions, as evidenced by weaker staining intensity and significantly lower IntDen scores compared to the IVM group. While the quantitative IntDen analysis of Bcl-2 and PCNA expressions in the liver sections revealed a marked decrease in the IVM-treated group compared to the control (Fig. 6C and D). In contrast, co-treatment with folic acid (IVM + FA) or vitamin B12 (IVM + Vit.B12) resulted in noticeably increased Bcl2 and PCNA expressions, as evidenced by stronger staining intensity and significantly higher IntDen scores compared to the IVM group.

### Histopathological and immunohistochemical findings of the kidney

**Table 5:** Quantitative analysis of integrated density (IntDen) values for Caspase-3, Bcl-2-associated X-protein (BAX), B-cell lymphoma 2(Bcl-2) and proliferating cell nuclear antigen (PCNA) expressions in the liver tissues (Mean ± S.D).

Tissues	Parameters	Ctrl-group	IVM-group	IVM + FA	IVM + Vit.B12
Liver	Caspase-3	0.05 ± 0.01^b^	15.25 ± 2.5^a^	2.64 ± 1.1^b^	2.26 ± 0.7^b^
	BAX	0.02 ± 0.01^b^	17.26 ± 0.6^a^	3.36 ± 0.2^b^	3.83 ± 1.0^b^
	Bcl-2	15.1 ± 2.6^b^	0.95 ± 0.10^c^	33.55 ± 1.7^a^	35.51 ± 7.5^a^
	PCNA	472.7 ± 23^a^	297 ± 40.9^b^	589 ± 3.60^a^	417.5 ± 15.9^a^

Values bearing different superscripts (a, b & c) in the same row differ significantly (*P* < 0.05).

Ctrl-group: control group, IVM group: Ivermectin group; IVM + FA: Ivermectin + Folic acid group, IVM + Vit.B12: Ivermectin + Vitamin B12 group.

**Fig. 7:**
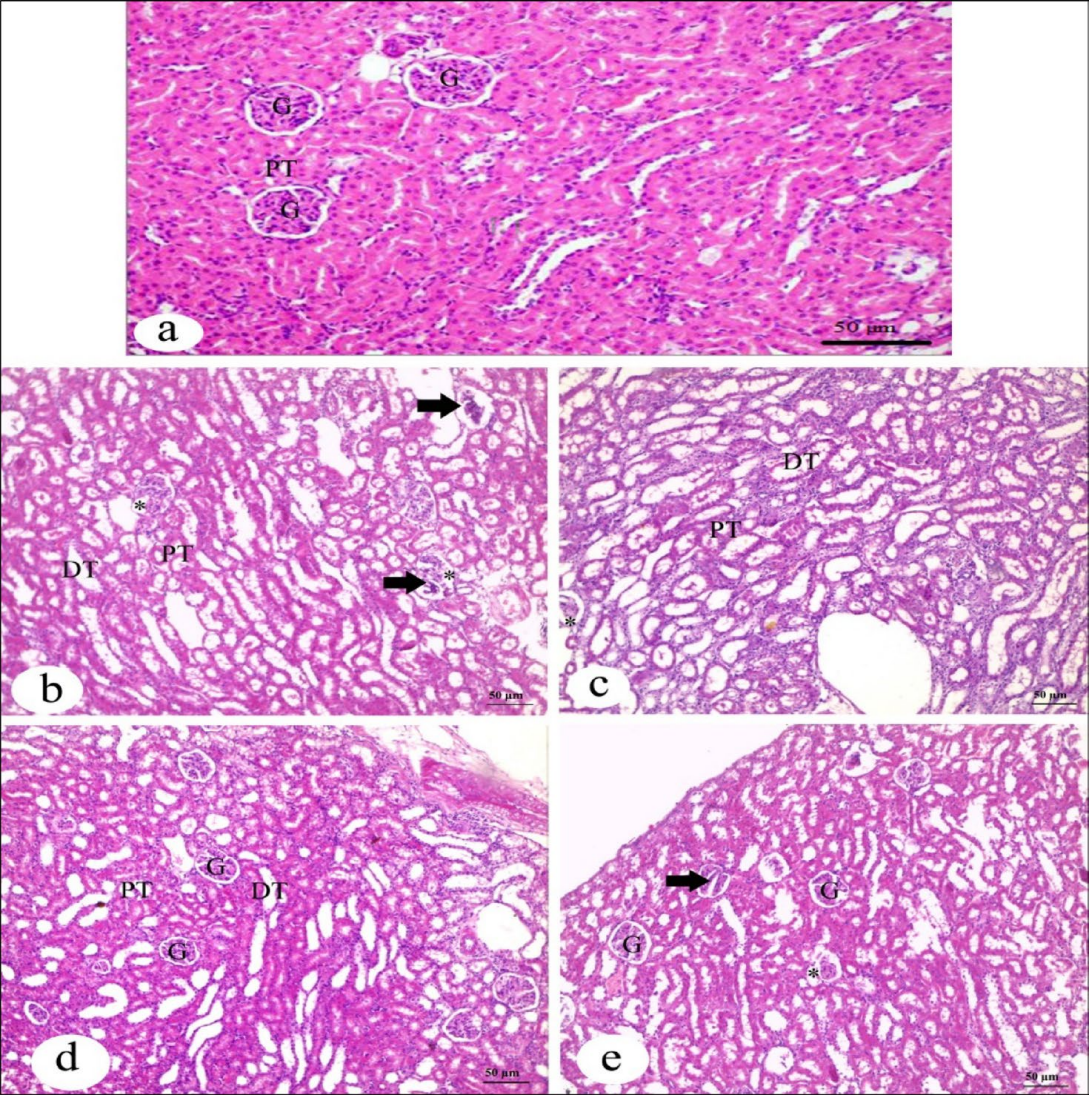
Photomicrograph of H&E-stained rat renal cortex of different experimental groups. (**a**) The control group shows normal histological structure, glomeruli (G), proximal convoluted tubule (PT) and distal convoluted tubule (DT). (**b**, **c**) IVM group shows different pathological lesions, segmented or destructed glomerulus (arrow), and dilated Bowman’s space (asterisk). (**d**) IVM + FA group and (**e**) IVM + Vit.B12 group-preserves their control character to a certain degree glomerulus (G), proximal convoluted tubule (PT), and distal convoluted tubule (DT). (H&E Bar = 50 μm).

The histological structure of the kidney appeared normal in the control group (Fig. 7). The IVM group showed different pathological lesions such as segmented and destructed glomerulus, and dilated Bowman’s space. The groups received FA or Vit.B12 with IVM almost showed normal structure similar to that of the control group.

**Fig. 8:**
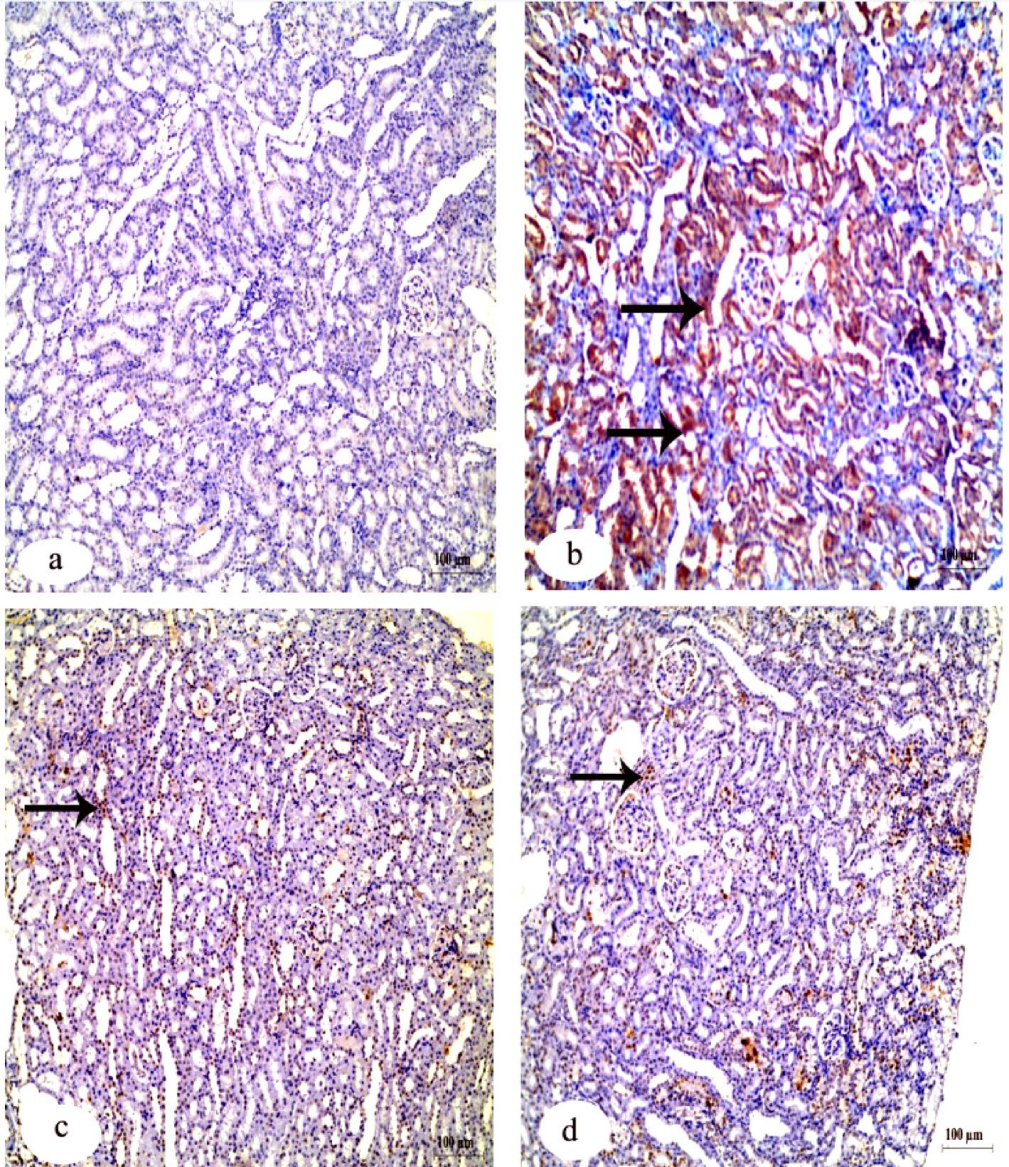
Immunohistochemical staining for cleaved caspase-3 expression in renal tissue sections of male albino rats. (**a**) Control group showing negative immunoreactivity. (**b**) IVM-treated group showing strong positive staining indicating increased apoptotic activity. (**c**) IVM + folic acid-treated group exhibiting moderate reduction in caspase-3 immunoreactivity compared to the IVM group. (**d**) IVM + vitamin B12-treated group showing minimal to absent immunoreactivity, approaching levels observed in the control group. Black arrows indicate positive reactions. Scale bar = 100 μm.

**Fig. 9:**
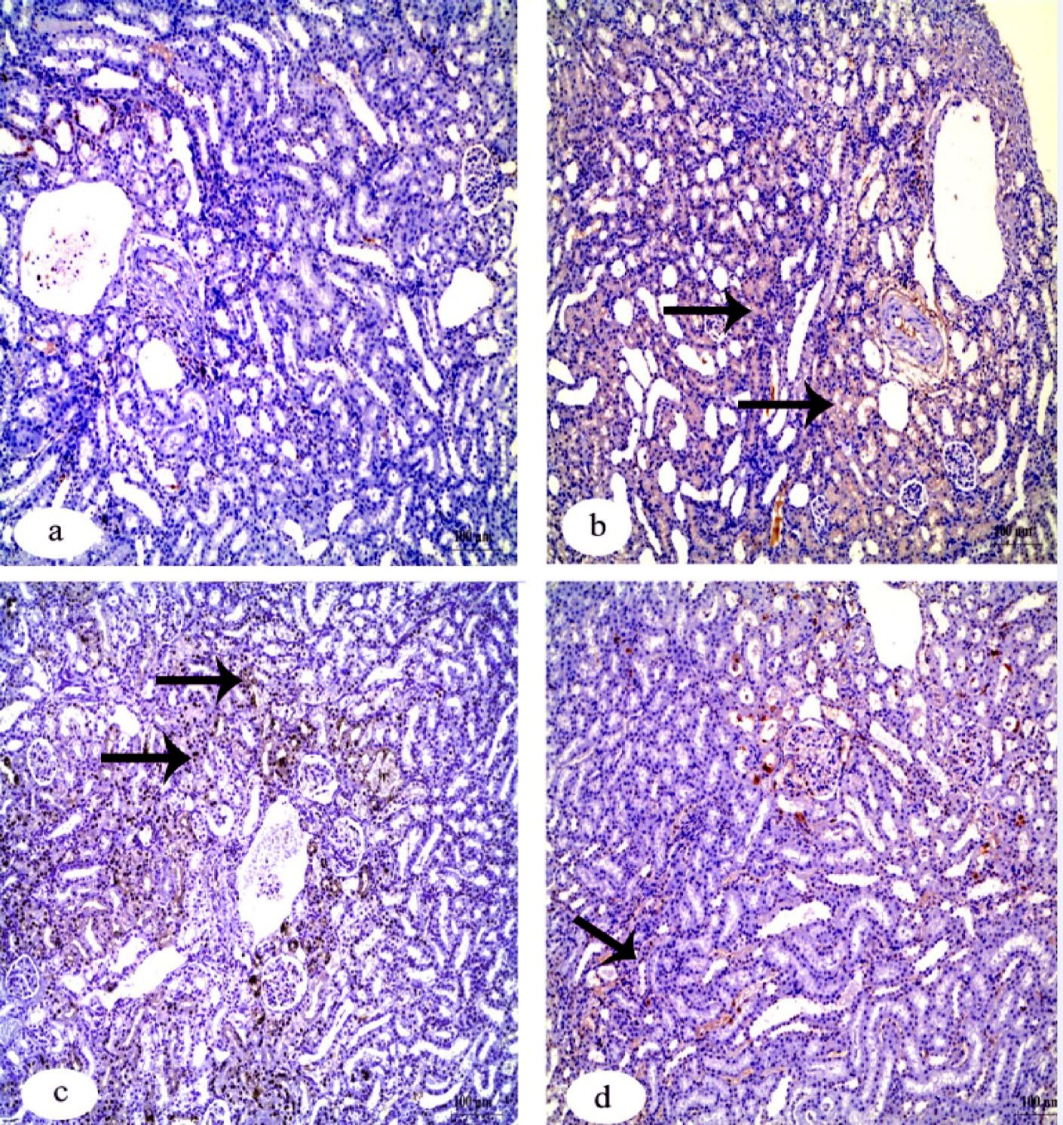
Immunohistochemical staining for Bax expression in renal tissue sections of male albino rats. (**a**) Control group showing negative immunoreactivity. (**b**) IVM-treated group showing strong positive staining indicating increased apoptotic activity. (**c**) IVM + folic acid-treated group exhibiting moderate reduction in Bax immunoreactivity compared to the IVM group. (**d**) IVM + vitamin B12-treated group showing minimal immunoreactivity, approaching levels observed in the control group. Black arrows indicate positive reactions. Scale bar = 100 μm.

**Fig. 10:**
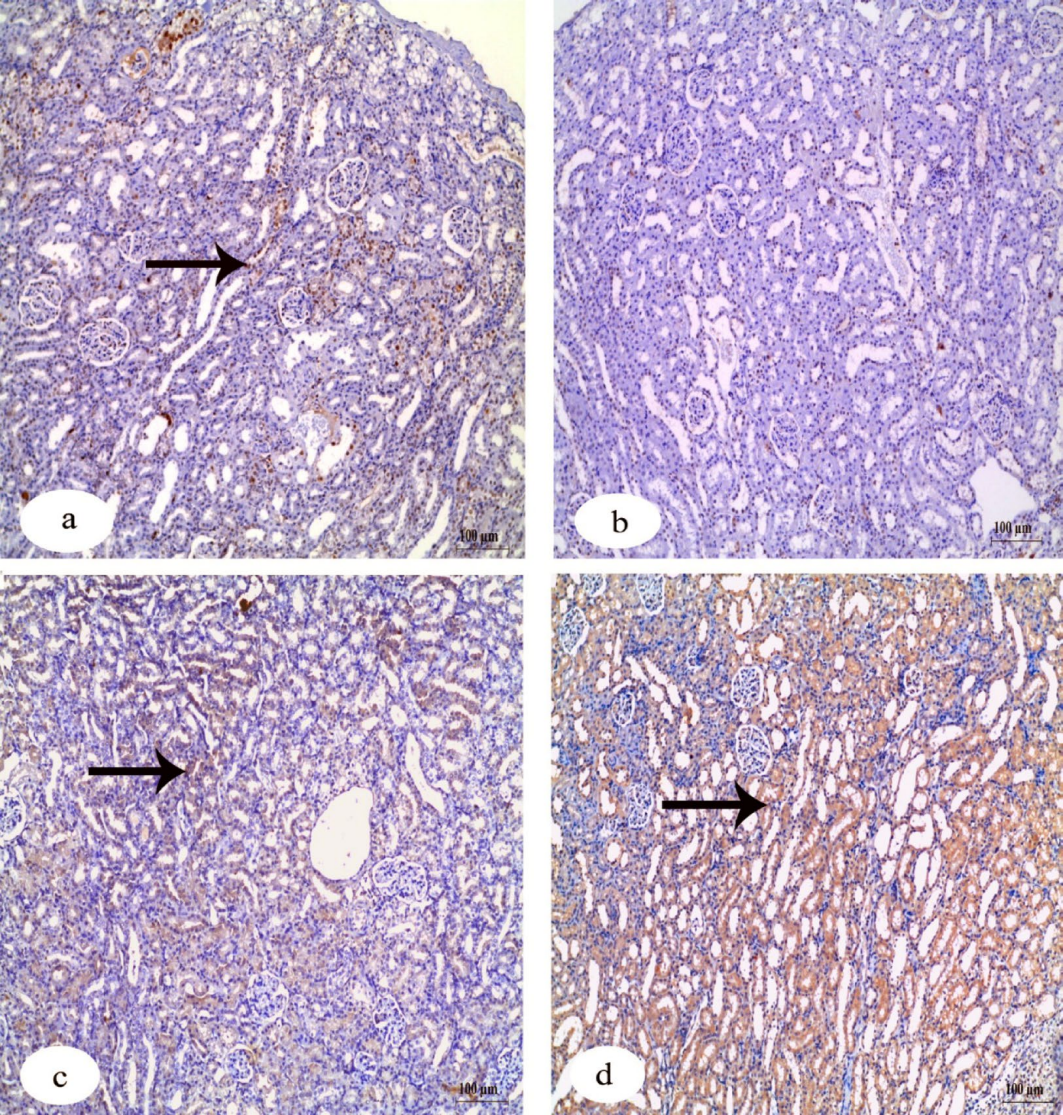
Immunohistochemical staining for Bcl-2 protein expression in renal tissue sections of male albino rats. (**a**) Control group showing strong positive Bcl-2 immunoreactivity. (**b**) IVM-treated group demonstrating a marked loss of Bcl-2 expression, indicating reduced anti-apoptotic activity. (**c**) IVM + folic acid-treated group showing partial restoration of Bcl-2 expression. (**d**) IVM + vitamin B12-treated group exhibiting positive Bcl-2 staining comparable to the control. Black arrows indicate positive reactions. Scale bar = 100 μm.

**Fig. 11:**
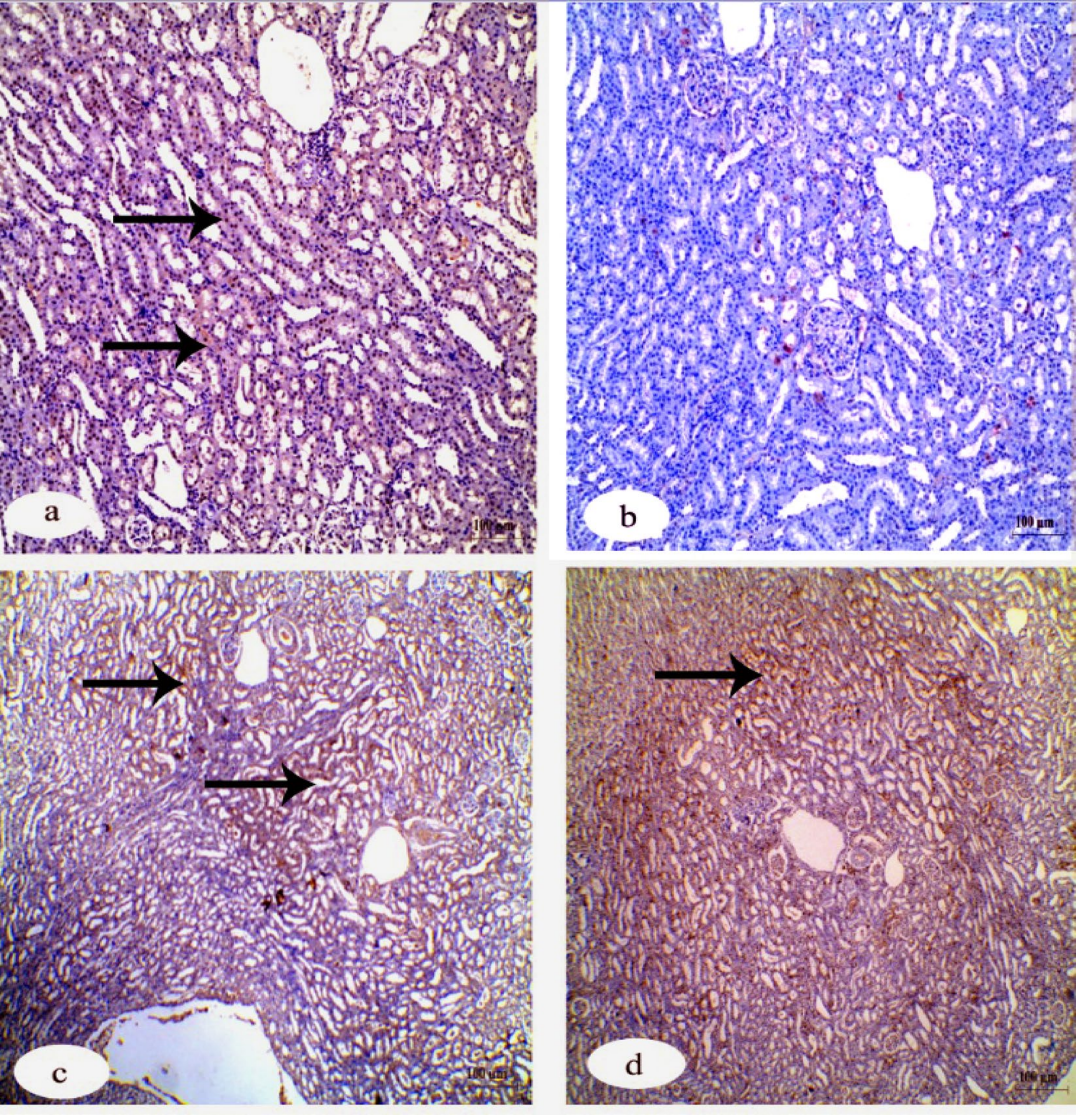
Immunohistochemical staining for PCNA expression in renal tissue sections of male albino rats. (**a**) Control group exhibiting strong positive PCNA immunoreactivity, indicating active cell proliferation. (**b**) IVM-treated group showing markedly reduced PCNA expression. (**c**) IVM + folic acid-treated group showing moderate restoration of PCNA-positive reaction. (**d**) IVM + vitamin B12-treated group showing PCNA expression levels comparable to the control group. Black arrows indicate positive reactions. Scale bar = 100 μm.

Immunohistochemistry investigation of caspase-3 in the kidney showed a negative reaction in the control group in contrast to the group that received IVM which showed a strong positive reaction. The other two groups that received FA and Vit.B12 showed weak reactions (Fig. 8). Immunohistochemistry investigation for BAX protein in the kidney showed negative reaction in the control group, while the group received IVM showed a positive reaction. The groups that received FA and Vit.B12 showed weak reactions (Fig. 9). Immunohistochemistry investigation for Bcl-2 and PCNA expressions showed positive reaction in the control group and negative reaction in the group received IVM. While, the groups were administrated FA or Vit.B12 showed positive reactions (Figs. 10 and 11, respectively).

**Fig. 12:**
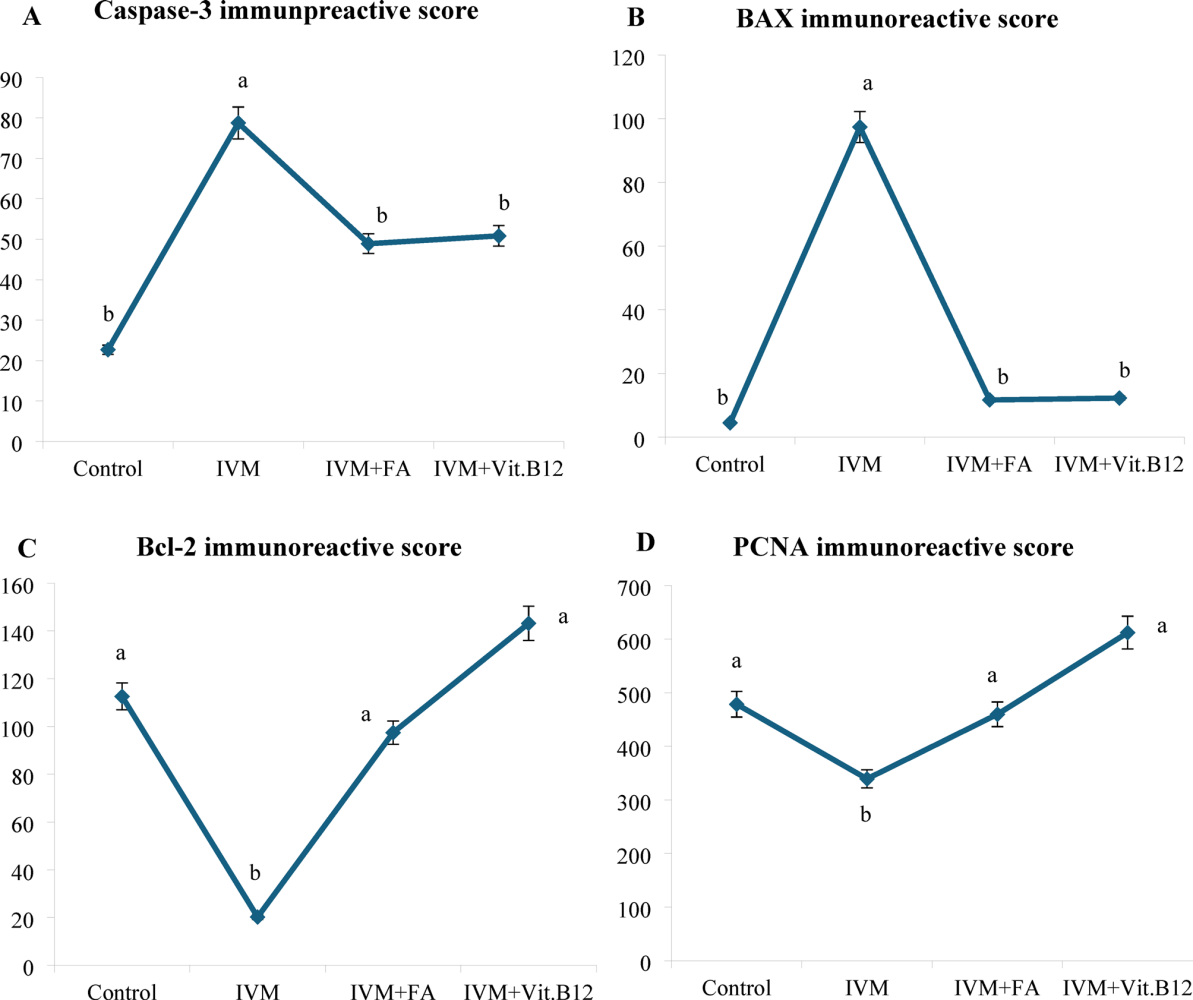
Immunoreactive score of (**A**) Caspase-3, (**B**) Bax, (**C**) Bcl-2 and (**D**) PCNA in kidney sections of male rats from different treatment groups. Values are expressed as means ± S.D. Values with different superscript (a and b) are significantly different at *P* < 0.05. Control: Control group; IVM: Ivermectin group; IVM + FA: Ivermectin + Folic acid group; IVM + Vit. B12: Ivermectin + Vit. B12 group.

Table 6 shows quantitative analysis of integrated density (IntDen) values for Caspase-3, BAX, Bcl-2 and PCNA expressions in the kidney tissues. Results of the IntDen analysis of cleaved caspase-3 and BAX expressions in the kidney sections revealed a marked increase in the apoptotic activity in the IVM-treated group compared to the control. The IVM group showed strong cytoplasmic immunoreactivity, as indicated by the intense brown staining and significantly elevated IntDen score (Fig. 12A and B). In contrast, co-treatment with folic acid (IVM + FA) or vitamin B12 (IVM + Vit.B12) resulted in noticeably reduced cleaved caspase-3 and Bax expressions, as evidenced by weaker staining intensity and significantly lower IntDen scores compared to the IVM group. While the quantitative IntDen analysis of Bcl-2 and PCNA expressions in the kidney sections revealed a marked decrease in the IVM-treated group compared to the control (Fig. 12C and D). In contrast, co-treatment with folic acid (IVM + FA) or vitamin B12 (IVM + Vit.B12) resulted in noticeably increased Bcl2 and PCNA expressions, as evidenced by stronger staining intensity and significantly higher IntDen scores compared to the IVM group.

**Table 6:** Quantitative analysis of integrated density (IntDen) values for Caspase-3, Bcl-2-associated X-protein (BAX), B-cell lymphoma 2(Bcl-2) and proliferating cell nuclear antigen (PCNA) expressions in the kidney tissues (Mean ± S.D).

Tissues	Parameters	Ctrl-group	IVM-group	IVM + FA	IVM + Vit.B12
Kidney	Caspase-3	22.68 ± 1.6^b^	78.76 ± 4^a^	48.9 ± 15.14^b^	50.84 ± 9.2^b^
	BAX	4.46 ± 1.3^b^	97.40 ± 11^a^	11.68 ± 2.6^b^	12.27 ± 2.8^b^
	Bcl-2	112.6 ± 5.3^a^	20.20 ± 1.3^b^	97.46 ± 2.3^a^	143.2 + 6.4^a^
	PCNA	478.5 ± 3.4^a^	339.3 ± 8.5^b^	459.7 ± 4.9^a^	612.1 ± 0.05^a^

Values bearing different superscripts (a & b) in the same row differ significantly (*P* < 0.05).

Ctrl-group: control group, IVM group: Ivermectin group; IVM + FA: Ivermectin + Folic acid group, IVM + Vit.B12: Ivermectin + Vitamin B12 group.

## Discussion

Nowadays, several diseases have emerged that are difficult to treat, such as COVID-19, which has significantly impacted human life and productivity^37^. However, a new direction in treatment has been explored through the use of high doses of ivermectin (IVM), which is still under clinical trials. Therefore, the current study is concerned with evaluating the potential harmful effects resulting from the use of high doses of IVM.

Several studies have used biochemical investigation and liver and kidney parameters as indicators to monitor changes that occur within the body^38,39^. In the current study, the increased levels of liver enzymes (ALT & AST) and the decreased level of ALB in the IVM group indicate liver cell damage induced by IVM. These changes in liver biomarkers confirm IVM toxicity. These findings are consistent with previous studies, which state that elevated liver enzyme levels and decreased ALB levels are indicators of liver injury^40,41^. Furthermore, recent study stated that IVM activity is not linked only with liver^42^ and suggesting a general damaged effect.

Folic acid and Vit.B12 supplied along with IVM treatment showed an improvement in the liver via decreasing ALT, AST, ALP and increasing albumin, TP specifically with FA treatment. Vit.B12 administration also provides similar positive results on liver except for increasing ALP in comparison with IVM group. However, it was reported that Vit.B12 change ALP levels depending on the concentration of the used Vit.B12^30^. In our study, the used dose of Vit.B12 was 50 mcg/kg which was higher than 3 mcg/kg, 25mcg that was used in other previous studies^30,43^. This may give an explanation for the rise of ALP levels in our study.

In the present work, IVM induced renal toxicity that was indicated by the significant increase in creatinine, urea, BUN and UA. It is well known that kidney responsible for BUN excretion and its damage leads to high levels of BUN in the blood^44^. In addition, kidney filters any wastes in the blood such as creatinine, UA and urea, so the damage on its cells disrupts this function and elevates the previously mentioned products. In the current study, it was observed that the levels of creatinine, urea, BUN and UA in the treated groups (FA and Vit.B12) was significantly reduced. Similarly, previous researches stated that FA administration cause reduction on the levels of creatinine^45,46^, urea^47^ and UA. Also, Vit.B12 administration was mentioned to cause similar decrease in the levels of these parameters^13,30^ in the same manner as FA.

In the present trial, IVM induced oxidative stress resulted from increasing MDA and decreasing SOD, CAT and TAC. Oxidative stress usually results from the imbalance between high free radicals and the decreased ability of antioxidants to get rid of it^48^. MDA is the end product of lipid peroxidation by free radicals, comes after cell membranes and lipoproteins damaging^49^. Also, IVM decreased SOD and CAT, which considered the enzymatic weapons of cells against free radicals (worked as antioxidants)^49^. In addition, TAC serves as antioxidant which asses the total antioxidant status in the biological sample specifically the amount of scavenged free radicals^50^. Several researches illustrated that oxidant/antioxidant profile play a role in induction and/or protection against different types of toxicity^51–54^. In contrast, it was observed that FA and Vit.B12 treatments counteracted the changes induced by IVM in oxidative markers evidencing the antioxidant activity of these vitamins. Recent research indicated that the Vit.B12 has a protective effect against IVM induced toxicity on blood parameters^55^.

Histological investigation of the changes in the liver revealed that the control group showed normal architecture with normal central veins and hepatocytes. This structure changed in the IVM group that showing dilatation of the blood vessels and multiple ballooned hepatocytes. Additionally, some inflammatory infiltration was also observed in the liver. These histopathological changes are similar to the findings reported by^56^, who confirmed the presence of inflammatory infiltrates in the liver after administration of IVM as a treatment against SARS-CoV-2 infection. Contrary, treatment with either FA or Vit.B12 restored a healthy liver architecture, highlighting the beneficial impact of these vitamins against the destruction caused by IVM.

The kidney was also histologically damaged after IVM administration, showing dilatation in Bowman’s space, alongside with other lesions and a destructed glomerulus. These findings are consistent with^57^. Fortunately, Vit.B12 reduced these lesions to resemble the normal structures of the kidney in the control group, aligning with Ozturk et al. (2022), where the efficacy of Vit. B12 in restoring normal renal histology after methotrexate administration was demonstrated. Similarly, FA proved its ability to reverse kidney damage, confirming its protective effects^30^.

Several studies have focused on investigating Caspase-3 protein (apoptotic marker) as an indicator of positive or negative health conditions in the body^20,58,59^. Immunohistochemical results of the current work confirmed the histological observations. Caspase-3 protein, which was negatively expressed in the liver and kidney of the control group, showed positive expression in the group that received IVM. It was previously announced that IVM induced the activation of both extrinsic and intrinsic caspase-dependent apoptotic pathways^60^. Additionally, a previous trial aimed to investigate the anticancer activities of IVM reported that increasing the concentration of IVM causes a collapse in mitochondrial membrane potential. This leads to an increase in the Bax/Bcl-2 ratio in the cytoplasm, which induces the release of cytochrome c from the mitochondria to the cytoplasm, activates caspase-9 and − 3, and ultimately triggers apoptosis^61^. This can give an explanation for the positive reaction of Caspase-3 protein in the IVM group. Treatment with FA and Vit.B12 resulted in a negative expression of caspase-3 and proving the efficacy of these vitamins in reducing the damaging effects of IVM.

Similarly, Bax investigation revealed negative expression in the liver and kidney of the control group and positive reaction in the IVM group, confirming the apoptosis caused by IVM. These findings resemble that was announced in the previous trial indicated that mechanistically, IVM significantly triggered ROS accumulation and inhibited the activation of NF-κB signaling pathway and increased the ratio of Bax/Bcl-2^62^. It is well known that Bax protein is a motivator for caspase-3 to induce apoptosis^63^. However, FA and Vit.B12 treatment in the present work succeeded to cause a relief of the bad effect of Bax as it was found negatively expressed.

Conversely, in the present study, Bcl-2 protein-which is known to play a role in cell regeneration-was positively expressed in the liver and kidney tissues of the control group, but negatively expressed in the IVM-treated group. However, the administration of vitamins such as FA and Vit.B12 restored the positive expression of Bcl-2. It has been reported that overexpression of this protein contributes to its anti-apoptotic function^64^.

Proliferating cell nuclear antigen (PCNA) is positively expressed during cell regeneration^65^. In this study, PCNA expressed positive expression in the liver and kidney of the control group, In contrast, the IVM group exhibited negative expression, indicating difficulty in cell regeneration, particularly due to suppression in the S phase of the cell cycle, which is responsible for DNA synthesis and replication^66^. While FA or Vit.B12 treatment restored the positive expression of PCNA, indicating their ability to promote regeneration. Previous research concerned with studying IVM long-term hazards indicated that the immunoreactivity of PCNA in testes tissues was mildly lower in the IVM group compared to the control groups^66^.

## Conclusion

Prolonged high-dose administration of ivermectin (IVM) in rats induced pronounced hepatotoxicity and nephrotoxicity, as shown by significant biochemical changes, histological damage, and elevated oxidative-stress markers. Folic acid and vitamin B12 exhibited potent antioxidant and anti-inflammatory effects that substantially mitigated IVM-induced toxicity. These results underscore the benefits of co-administering FA and vitamin B₁₂ with IVM to preserve its therapeutic efficacy-including enhanced food security-while minimizing adverse effects on the liver and kidneys. Although compelling, these findings also highlight the need for future research to define optimal dosing, assess translational relevance in humans, and ensure long-term safety in clinical protocols.

## Data Availability

All data generated or analyzed during this study are included in this published article.
